# Chrdl1-mediated BMP4 inhibition disrupts the balance between retinal neurons and Müller Glia

**DOI:** 10.1038/s41420-024-02129-6

**Published:** 2024-08-17

**Authors:** Dongmei Liu, Zeyuan Pu, Baige Li, Gao Tan, Ting Xie, Yin Shen

**Affiliations:** 1 Eye Center, Renmin Hospital of Wuhan University, Wuhan, Hubei 430060 P. R. China; 2 Department of Anesthesiology & Center for Brain Science, The First Affiliated Hospital of Xi’an Jiaotong University, Xi’an, Shanxi 710061 P. R. China; 3 Division of Life Science, State Key Laboratory of Molecular Neuroscience, the Hong Kong University of Science and Technology, Clear Water Bay, Hong Kong, 999077 P. R. China; 4 Frontier Science Center for Immunology and Metabolism, Medical Research Institute, Wuhan University, Wuhan, Hubei 430060 P. R. China

**Keywords:** Developmental neurogenesis, Embryonic induction, Experimental models of disease

## Abstract

Chordin-like 1 (CHRDL1) is a secreted protein that serves as an endogenous antagonist of bone morphogenetic proteins (BMPs). In the developing retina, *Bmp4* has been demonstrated to be essential for sustaining the proliferation of progenitor cells and facilitating the differentiation of glial cells. Despite these efforts, the precise effects of *Bmp4* inhibition on the developing retina are yet to be fully understood. We sought to address this question by overexpressing *Chrdl1* in the developing retina. In this study, we explored the impact of *Bmp4* inhibition on the developing mouse retina by conditionally overexpressing the *Bmp4* inhibitor *Chrdl1*. Initially, we characterized the expression patterns of *Bmp4* and *Chrdl1* in the developing mouse retina from E10.5 to P12.5. Additionally, we utilized various molecular markers to demonstrate that *Bmp4* inhibition disrupts both neuronal and Müller glial differentiation in the developing mouse retina. Moreover, through the application of RNA-seq analysis, distinctively expressed retinal genes under the modulation of *Bmp4* signaling were discerned, encompassing the upregulation of Id1/2/3/4 and Hes1/5, as well as the downregulation of Neurod1/2/4 and Bhlhe22/23. Lastly, electroretinogram (ERG) and optomotor response (OMR) assays were conducted to illustrate that *Bmp4* inhibition impairs the functional connectivity of various cells in the retina and consequently affects visual function. Collectively, this study demonstrates that inhibiting *Bmp4* promotes the differentiation of retinal neurons over Müller glia by activating the expression of genes associated with neuron specification. These findings offer molecular insights into the role of *Bmp4* signaling in mammalian retinal development.

## Introduction

Bone morphogenetic proteins (BMPs) constitute a remarkably conserved and diverse protein family, crucially contributing to the development and organization of the central nervous system (CNS) [1]. A multitude of studies have demonstrated the pivotal role of the BMP signaling pathway in the initiation and specification of eye development. This encompasses the induction of optic vesicle, optic cup, anterior segment, and lens formation in the early stages, as well as its involvement in retinogenesis during the later stages [2–5]. In the early stages of eye development, the BMP family (*Bmp2, Bmp4, and Bmp7*) is predominantly expressed in the optic nerve primordium and optic vesicle. Following the formation of the optic cup at E10.5, BMP expression becomes distributed in various regions. In the chicken retina, *Bmp7* is mainly localized in the lens ectoderm [6], while *Bmp2* is concentrated in the outer neuroepithelial layer of the optic cup, playing a regulatory role in the formation of RPE [7]. Additionally, *Bmp4* is primarily expressed in the inner neuroepithelial layer of the optic cup [8], participating in the regulation of neural retina formation. However, the distribution and function of *Bmp4* in mammalian retina development remain not well understood.

The BMP4 gene, located on 14q22-q23, is strongly associated with ocular malformations [9]. Heterozygous *Bmp4*^+/−^ mice exhibit anophthalmia-microphthalmia, failure of lens induction, anterior segment dysgenesis, and retinal and optic nerve aplasia [10–12]. Similarly, clinical studies have revealed that individuals with anophthalmia or microphthalmia, combined with sclerocornea, were found to harbor a nonsense mutation in *Bmp4* [13]. Furthermore, maintaining an optimal level of *Bmp4* signaling is crucial for proper growth along the dorso-ventral axis of the optic cup, as excessive activation can result in reduced ocular growth, leading to small and misshapen eyes [14]. Given the indispensable role of *Bmp4* in the early stages of eye development, previous studies have investigated its impact on retinal cell differentiation following optic cup formation in the late stage of retinal development [15]. However, the precise role of *Bmp4* in the morphological evolution and functional development of various retinal neurons remains to be fully elucidated.

The Human Protein Atlas (http://www.proteinatlas.org/) reveals that chordin-like 1 (Chrdl1) is the most prominently expressed inhibitor of BMPs signaling in the human and murine retina. Studies have demonstrated that *Chrdl1* can alter the fate of progenitor cells by inhibiting the BMPs signaling pathway, thereby promoting the differentiation of neural progenitor cells into the neuronic lineage within the central nervous system [16]. Clinical studies have reported that dysregulation of CHRDL1-BMP4 antagonism leads to X-linked megalocornea in humans [17]. Based on these findings, we hypothesize that *Chrdl1* and *Bmp4* play a role in regulating cell differentiation during retinal development.

In this study, we initially explored the expression patterns and localization of *Bmp4* and *Chrdl1* in the retina. Subsequently, a mouse model with specific overexpression of *Chrdl1* in the retina was established using the cre-loxp system. We then investigated the impact of inhibiting *Bmp4* signaling on various aspects of retinal development, encompassing retinal tissue structure and visual function. Finally, through RNA-seq analysis, we unraveled the mechanisms underlying the actions of *Bmp4* and *Chrdl1*, shedding light on their roles in retinal differentiation. This study provides novel insights into the understanding of *Chrdl1*-mediated *Bmp4* signaling in retinal development.

## Results

### Temporal and spatial expression patterns of Bmp4 in retinal development

**Fig. 1:**
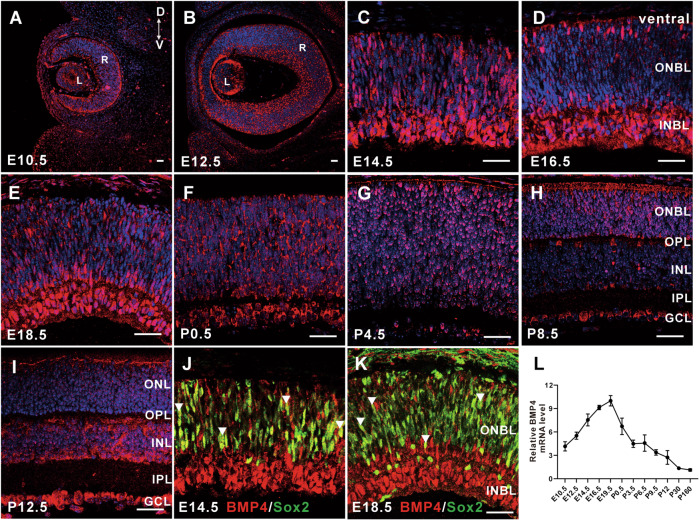
Spatiotemporal expression pattern of *Bmp4* during mouse retinal development. **A**–**I** The *Bmp4* immunostaining of the retinal sections at the stage of the indicated developmental stages. **J**, **K** The section in (**C**–**E**) was co-labeled with an anti-Sox2 antibody. **L** Temporal expression levels of *Bmp4* from embryonic to adult stages during retinal development as determined by qRT-PCR analysis. Results are presented as the mean ± SEM (*n* = 3). Scale = 20 μm. Abbreviations: L lens, R retina, GCL ganglion cell layer, INBL inner neuroblastic layer, INL inner nuclear layer, IPL inner plexiform layer, ONBL outer neuroblastic layer, ONL outer nuclear layer, OPL outer plexiform layer.

While a previous study has reported on the expression pattern of BMP receptors in retinal development [18], the comprehensive investigation of *Bmp4* expression patterns in this context remains unexplored. Immunofluorescence was conducted to characterize the spatial and temporal expression patterns of *Bmp4* in the mouse retina. As depicted in Fig. 1A, the expression of *Bmp4* in the mouse retina exhibited a weaker signal at the embryonic stage (E) 10.5. By E12.5, the expression of *Bmp4* became more pronounced compared to E10.5 (Fig. 1B). From E14.5 to E18.5, *Bmp4* expression significantly increased in the entire population of retina progenitor cells, especially in the inner neuroblastic layers (INBL), while remaining robust in lens cells (Fig. 1C–E). From postnatal (P) 0.5 to mature retinas, *Bmp4* gradually localizes to the ganglion cell layer (GCL) and the innermost region of the inner nuclear layer (INL), while its expression diminishes in the outer nuclear layer (ONL) (Fig. 1F–I). Additionally, our findings indicate that *Bmp4* is predominantly expressed in Sox2+ retinal progenitor cells (RPCs) at E14.5 and E18.5 (Fig. 1J, K). In alignment with the immunofluorescence signal profile, *Bmp4* mRNA expression maintains a high level during the embryonic stage, reaching its peak at E19.5, and subsequently decreases gradually with the development and maturation of the retina, persisting until P160 (Fig. 1L). These findings elucidated the dynamic nature of *Bmp4* expression within the murine retina, revealing distinct spatiotemporal patterns of gene transcription. *3.2. Temporal l and spatial expression patterns of Chrdl1 expression during mouse retinal development.*

**Fig. 2:**
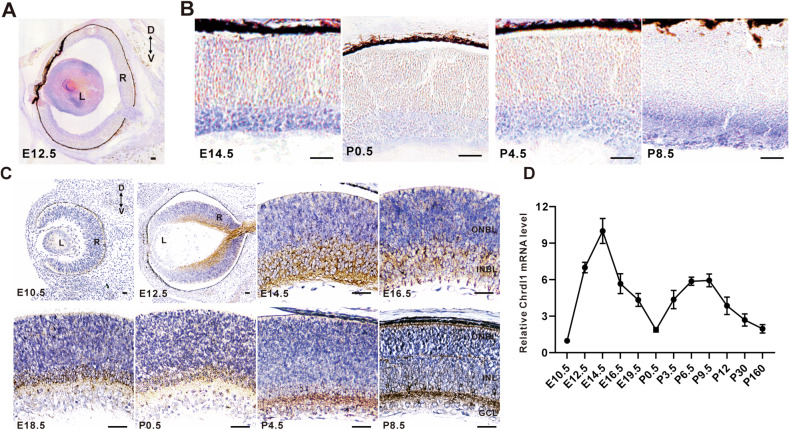
Spatiotemporal expression pattern of *Chrdl1* during mouse retinal development. **A**, **B** In situ hybridization of retinal sections of the target period with a *Chrdl1* probe. **C** Retinal sections from the indicated developmental stages were immune stained with an anti-Chrdl1 antibody and counterstained with DAPI. **D** By qRT-PCR analysis, the temporal expression levels of *Chrdl1* during retinal development from embryonic to adult stages were determined. Results are presented as the mean ± SEM (*n* = 3). Scale = 20 μm. Abbreviations: L lens, R retina, GCL ganglion cell layer, INBL inner neuroblastic layer, INL inner nuclear layer, ONBL outer neuroblastic layer, ONL outer nuclear layer.

*Chrdl1* antagonizes the function of *Bmp4* by binding to the *Bmp4* ligand, blocking its interaction with the receptor. Therefore, we employed situ hybridization and immunohistochemistry to investigate the temporal and spatial expression patterns of *Chrdl1* during mouse retinal development. As depicted in Fig. 2A, the expression of *Chrdl1* RNA was notably diminished in the retina, while it exhibited an elevated level in the lens at E12.5. Following a dynamic temporal progression, *Chrdl1* RNA expression became confined to the inner neuroblastic layers (INBL) from E14.5 to P8.5 (Fig. 2B). The expression pattern of *Chrdl1* protein closely mirrored its mRNA expression. Specifically, at E10.5, *Chrdl1* expression was absent in the mouse retina, but discernible in the retina and restricted to the INBL at E12.5-E18.5. During early postnatal stages (P0.5, P4.5), *Chrdl1* expression was confined to the inner layers of the retina. By P8.5, it became restricted to the inner nuclear layer (INL) and ganglion cell layer (GCL) (Fig. 2C). Quantitative real-time polymerase chain reaction (qRT-PCR) results mirrored the profiles observed in RNA in situ hybridization signals, indicating that *Chrdl1* expression reached higher levels from E12.5 to E19.5 and subsequently maintained lower levels at least until P160 (Fig. 2D). These findings suggest that the expression pattern of *Chrdl1* in the mouse retina is characterized by temporal and spatial variations.

### Effect of Chrdl1 overexpression on the formation of retinal non-photoreceptor cells

In pursuit of this objective, we conducted targeted modifications to the ubiquitously expressed Rosa26 locus to facilitate the transcriptional expression of human *Chrdl1*. LoxP-flanked TpA stop signals were strategically introduced to mitigate the constitutive expression of the *Chrdl1* gene. Subsequently, the introduction of a Six3-Cre transgene engendered a murine model wherein the expression of human *Chrdl1* was selectively activated in the retina (designated as Rosa26-hChrdl1Six3-Cre, henceforth denoted as R26-hChrdl1), all while developing against an otherwise unaltered genetic backdrop. As anticipated, homozygous mice (Six3^pos^hChrdl1^+/+)^, characterized by the presence of two copies of the hChrdl1 gene, manifested discernibly heightened levels of hChrdl1 and a significant decrease in *Bmp4* levels within the retina in comparison to their heterozygous counterparts (Six3^pos^hChrdl1^+/-^) and control (Six3^neg^hChrdl1^+/+^) (Fig. S1C and S2). In addition, we conducted additional experiments to consider the possible involvement of other BMP family ligands. Nevertheless, in contrast, only the expression of Bmp4 was markedly downregulated (Fig. S3).

**Fig. 3:**
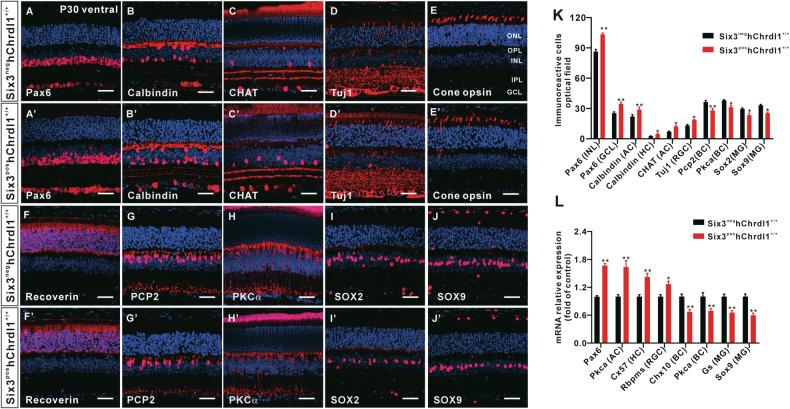
Effect of *Chrdl1* overexpression on the formation of retinal cell types. **A**–**J**, **A**’–**J**’ Sections from P30 Six3^neg^hChrdl1^+/+^ and Six3^pos^hChrdl1^+/+^ retinas were immune-stained with antibodies against the indicated cell type-specific markers and weakly counterstained with nuclear DAPI. Red represents Pax6 (RGCs, ACs), Calbindin (ACs, HCs), Chat (AC), Tuj1 (RGCs), Cone opsin (Cone), Recoverin (Photoreceptor cell), Pcp2 (BCs), Pkcα(BCs), Sox2 (MG) and Sox9 (MG), respectively. Scale = 20 μm. **K** Quantitation of the retinal cells for each specific marker per retina section. **L** The expression of the selected retinal cell-related genes at P30 in Six3^neg^hChrdl1^+/+^ and Six3^pos^hChrdl1^+/+^ retinas were determined by qRT-PCR analysis. Results are presented as the mean ± SEM (*n* = 3). ^*^p < 0.01, ^**^p < 0.05^.^ Abbreviations: AC Amacrine Cell, BC bipolar cell, HC horizontal cell, MG Müller glia cells, RGC retinal ganglion cell.

To elucidate the impact of inhibiting the BMP signaling pathway on the differentiation of distinct cell types and subtypes within the Six3^pos^hChrdl1^+/+^ retina, we characterized a spectrum of cell subtypes through immunofluorescence staining at postnatal day 30 (P30). In the Six3^pos^hChrdl1^+/+^, a notable increase in the population of Pax6^+^ cells (including amacrine and ganglion cells) was observed in the inner nuclear layer (INL) and ganglion cell layer (GCL) (Fig. 3A, A’). Correspondingly, a concordant elevation was noted in the numbers of Calbindin^+^ (amacrine cells) and Chat^+^ cells (amacrine cells), as depicted in Fig. 3B, C, along with their respective subfigures (Fig. 3B’, C’). Likewise, a marked augmentation was evident in the counts of Calbindin^+^ (horizontal cells) and Tuj1^+^ (ganglion cells) cells within the Six3^pos^hChrdl1^+/+^ retinas (Fig. 3B, B’, D, and D’). Importantly, no discernible difference was identified in the population of photoreceptor cells between Six3^pos^hChrdl1^+/+^ and Six3^neg^hChrdl1^+/+^ retina (Fig. 3E, F, E’, and F’). Concurrently, there was a notable reduction in the population of PKCα+ rod-bipolar cells and Pcp2+ on-bipolar cells within the Six3^pos^hChrdl1^+/+^ retina (Fig. 3G, H, G’, and H’). Müller cells assume a pivotal role in retinal homeostasis, overseeing the maintenance of both morphology and structural integrity. Our observations discerned a statistically significant decrease in the abundance of Sox2^+^ and Sox9^+^ Müller cells within the Six3^pos^hChrdl1^+/+^ retina (Fig. 3I, J, I’, and J).

Quantitative assessment of immunoreactive cell populations revealed a significant augmentation in the number of early-born cell types, including Pax6^+^/Calbindin^+^ amacrine, Calbindin^+^ horizontal, and Chat^+^/Tuj1^+^ cells, within the Six3^pos^hChrdl1^+/+^ retina. Specifically, an increase of 32%, 26%, 24%, 42%, and 29%, respectively, was observed in comparison to the Six3^neg^hChrdl1^+/+^ retina. Conversely, late-born cell types in the Six3^pos^hChrdl1^+/+^ retina, namely PKCα^+^ rod bipolar, Pcp2^+^ on bipolar, Sox2^+^ Müller cells, and Sox9^+^ Müller cells, exhibited a decrease of 21%, 30%, 26%, and 31%, respectively, as compared to the Six3^neg^hChrdl1^+/+^ retina (Fig. 3K). Quantitative real-time polymerase chain reaction (QRT-PCR) was employed to corroborate the mRNA expression changes of specific markers corresponding to each cell type following Chrdl1 overexpression. The outcomes demonstrated congruence with the cell count results (Fig. 3L). In summary, our experimental findings indicate that the conditional overexpression of Chrdl1 results in an augmented abundance of early-born cell types (retinal ganglion cells, horizontal cells, and amacrine cells), concomitant with a diminished presence of later-born cell types (bipolar cells and Müller glial cells).

### Chrdl1 overexpression alters the specification of non-photoreceptor cells in the early stage of retinal development

**Fig. 4:**
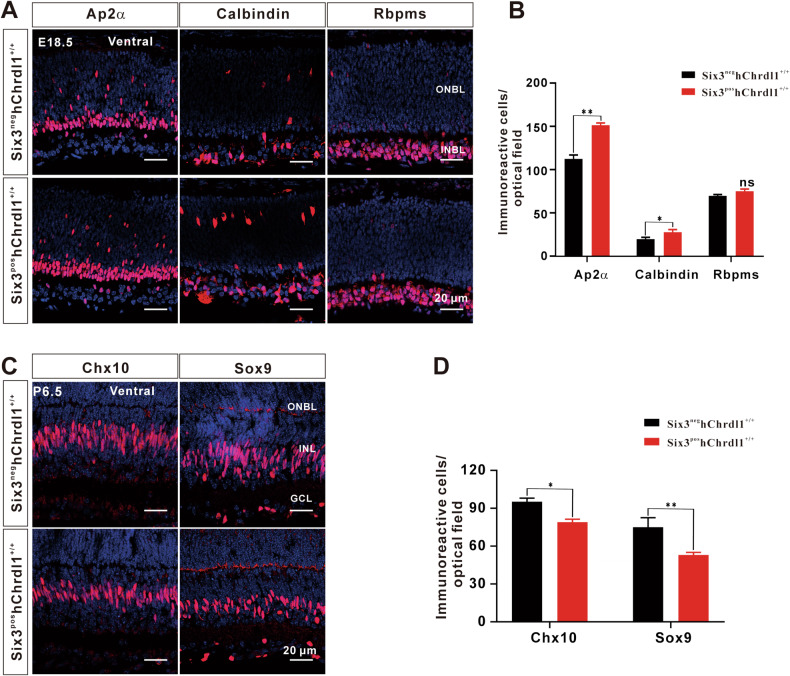
Effect of *Chrdl1* overexpression on the generation of different retinal cell types. **A** Sections E18.5 Six3^neg^hChrdl1^+/+^ and Six3^pos^hChrdl1^+/+^ retinas were immune-stained with antibodies against the indicated cell type-specific markers and weakly counterstained with nuclear DAPI. Red represents Ap2α (ACs), Calbindin (ACs, HCs), Rbpms (RGCs) respectively. Scale = 20 μm. **B** Quantitation of cells that are immunoreactive for the indicated cell type-specific markers in E18.5 (Ap2α, Calbindin, Rbpms) *Chrdl1*^f1/f1^ and *Chrdl1*^Six3-KI^ retinas. **C** Sections P6.5 Six3^neg^hChrdl1^+/+^ and Six3^pos^hChrdl1^+/+^ retina were immune-stained with antibodies against the indicated cell type-specific markers and weakly counterstained with nuclear DAPI. Red represents Chx10 (BCs) and Sox9 (MG) respectively. Scale = 20 μm. **D** Quantitation of cells that are immunoreactive for the indicated cell type-specific markers in P6.5 (Chx10, Sox9) *Chrdl1*^f1/f1^ and *Chrdl1*^Six3-KI^ retinas. Results are presented as the mean ± SEM (*n* = 3). ^*^p < 0.01, ^**^p < 0.05. Abbreviations: RGC, retinal ganglion cell; AC, Amacrine Cell; HC, horizontal cell; BC, bipolar cell; MG, müller glia cells.

In the early stages of retinal development, retinal progenitor cells undergo differentiation into mature retinal neurons under the influence of transcription factors [19]. In this study, we investigated the development of non-photoreceptor cells in Six3^neg^hChrdl1^+/+^ and Six3^pos^hChrdl1^+/+^ retinas. Comparative analysis with the Six3^neg^hChrdl1^+/+^ group revealed a significant increase in the number of amacrine cells (Ap2α^+^ or Calbindin^+^) and horizontal cells (Calbindin^+^) in the Six3^pos^hChrdl1^+/+^ group at E 18.5. Conversely, the count of ganglion cells, immunoreactive for Rbpms, showed no significant change between Six3^neg^hChrdl1^+/+^ and Six3^pos^hChrdl1^+/+^ retinas at P 6.5 (Fig. 4A, B).

Furthermore, there was a notable reduction in the number of Chx10^+^ bipolar cells and Sox9^+^ Müller glial cells in the Six3^pos^hChrdl1^+/+^ group at P 6.5 (Fig. 4C, D). To provide additional insights into the roles of Chrdl1 and Bmp4 in early-stage retinal development, we labeled E 14.5 Six3^neg^hChrdl1^+/+^ and Six3^pos^hChrdl1^+/+^ retinal progenitor cells with Chx10 and Sox2 antibodies. Comparative analysis with Six3^neg^hChrdl1^+/+^ revealed a significant decrease in the number of Chx10^+^ or Sox2^+^ cells (representing retinal progenitor cells) in the Six3^pos^hChrdl1^+/+^ group (Fig. S4). These results suggest that Chrdl1 is essential for the specification of amacrine, horizontal, bipolar, and Müller glial cells, while its role in the specification of ganglion cells appears to be limited.

### Chrdl1 overexpression impaired ERG responses and visual acuity

**Fig. 5:**
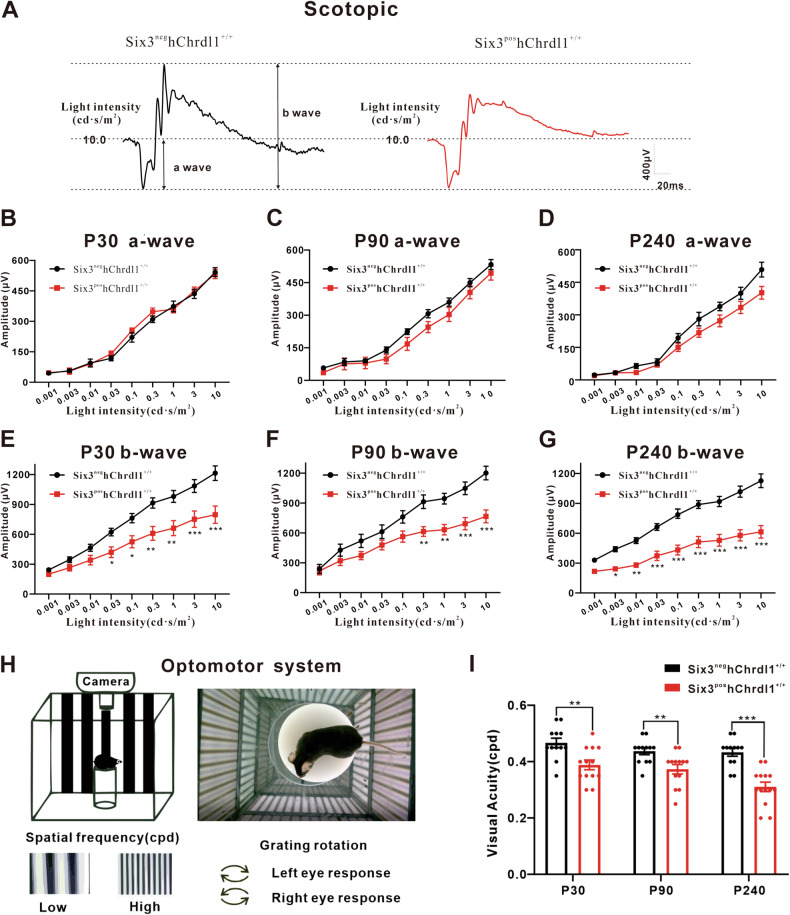
*Chrdl1* overexpression causes deficit in electroretinogram (ERG) responses and visual acuity. **A** Representative ERG wave forms of 10.0 light intensity (cd·s/m^2^) from dark-adapted Six3^neg^hChrdl1^+/+^ and Six3^pos^hChrdl1^+/+^ mouse aged P30. **B**–**D** The amplitudes of the scotopic ERG a-waves elicited from mice were recorded at P30, P90 and P240. **E**–**G** The amplitudes of the scotopic ERG b-waves elicited from mice were recorded at P30, P90 and P240. **H** Schematic illustrating the OMR testing apparatus and procedures. A mouse is placed freely on a platform, and the stimulus gratings are displayed on the LED screens surrounding the mouse (left). A mouse is tracking the moving grating on the top of the platform (right). **I** The visual acuity threshold in the scotopic OMR was measured from Six3^neg^hChrdl1^+/+^ and Six3^pos^hChrdl1^+/+^ mice aged at P30, P90, and P240. Results are presented as the mean ± SEM (*n* = 12). ^*^p < 0.01, ^**^p < 0.05^, ***^p < 0.001.

To assess the impact of Chrdl1 overexpression on the visual function of mice, electroretinogram (ERG) responses were recorded at postnatal days 30 (P30), 90 (P90), and 240 (P240) in both Six3^neg^hChrdl1^+/+^ and Six3^pos^hChrdl1^+/+^ mice. Representative waveforms for scotopic ERG responses at 10.0 cd·s/m2 are illustrated in Fig. 5A, indicating decreased b-wave amplitudes in Six3^pos^hChrdl1^+/+^ mice. Correspondingly, scotopic ERG responses at 30.0 cd·s/m2 are presented in Fig. S5A. Under dark-adapted conditions, for low flash intensities (0.003–0.03 cd·s/m2), ERG responses were predominantly characterized by the b-wave, with consistently lower response amplitudes observed in mutant mice compared to Six3^neg^hChrdl1^+/+^ animals. Notably, the a-wave exhibited no discernible changes in either light-adapted or dark-adapted environments between Six3^neg^hChrdl1^+/+^ and Six3^pos^hChrdl1^+/+^ mice at P30, P90, and P240 (Fig. 5B–D, Fig. S5B–D). Contrary to the stability of the a-wave, the amplitudes of the b-wave, primarily driven by the activity of Rod bipolar cells, exhibited an ~30% decrease in Six3^pos^hChrdl1^+/+^ mice at P30, P90, and P240 (Fig. 5E–G). In alignment with these findings, the b-wave amplitudes of Six3^pos^hChrdl1^+/+^ mice in light-adapted ERG were reduced by about 20% compared to the Six3^neg^hChrdl1^+/+^ group (Fig. S5E–G). The outcomes of electroretinogram (ERG) responses serve as indicative measures of the functional connectivity within various retinal cell populations. Specifically, alterations in the a-wave predominantly stem from changes in photoreceptor cells, while modifications in the b-wave are primarily attributed to alterations in the functional connectivity of diverse cells in the inner nuclear layer [20]. These changes may be intricately linked to variations in both the photoreceptor and non-photoreceptor cell populations.

Furthermore, the optomotor response (OMR) serves as a valuable tool for evaluating animal visual function, inducing eye movements through light stimulation at different frequencies (Fig. 5H). Our findings reveal a significant reduction in visual function in Six3^pos^hChrdl1^+/+^ mice at postnatal days 30, 90, and 240, respectively (Fig. 5I). Collectively, these results indicate that the conditional overexpression of Chrdl1 primarily impairs the visual acuity and ERG responses of the mice.

### The overexpression of Chrdl1 induces a cell fate switch during retinal development

**Fig. 6:**
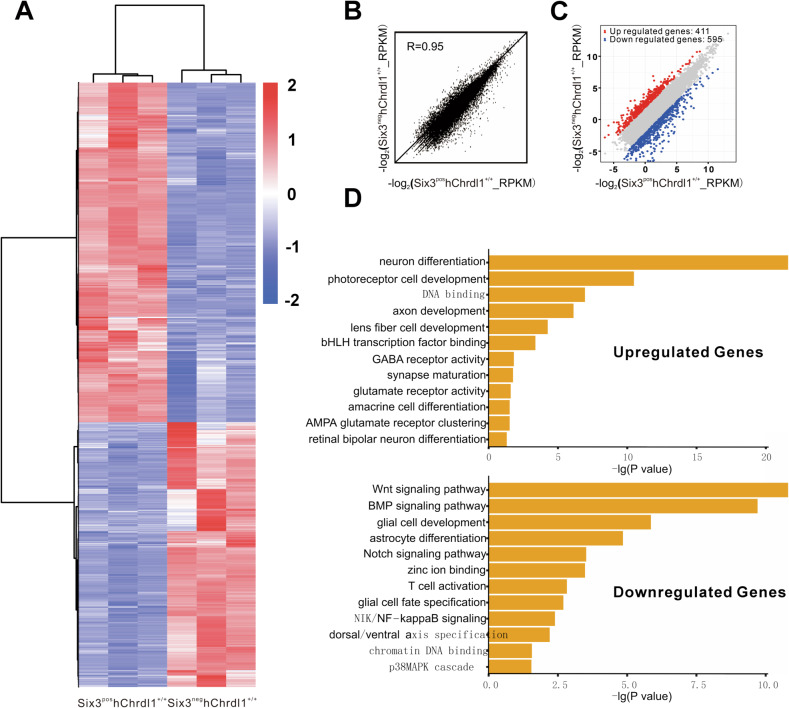
RNA-seq analysis reveals the differentially expressed genes in Six3^pos^hChrdl1^+/+^ retina at P0.5. **A** Cluster analysis presented a large group of significantly upregulated genes and a smaller group of significantly downregulated genes in the Six3^pos^hChrdl1^+/+^ retinas. **B** The scatter plot of global gene expression profiles and the Spearman correlation coefficient were indicated. The levels of gene expression (reads per kilobase per million reads) were depicted in log10 scale. The diagonal line represented equal expression levels in the Six3^neg^hChrdl1^+/+^ and Six3^pos^hChrdl1^+/+^ retinas. **C** Scatter plot of significantly upregulated (red) and downregulated (blue) genes (fold change ≥ 2.0 and *P* < 0.05) between the Six3^neg^hChrdl1^+/+^ and Six3^pos^hChrdl1^+/+^ retinas. **D** Gene ontology (GO) terms that related to the retina of upregulated and downregulated genes set in Six3^pos^hChrdl1^+/+^ retinas compared to Six3^neg^hChrdl1^+/+^ retinas.

To delve deeper into the functional and mechanistic aspects of the Chrdl1-mediated Bmp4 signal in retinal development, we conducted RNA-seq analysis on the retinas of Six3^neg^hChrdl1^+/+^ and Six3^pos^hChrdl1^+/+^ mice at postnatal day 0.5 (P0.5). As anticipated, a significant disparity in gene expression profiles between Six3^neg^hChrdl1^+/+^ and Six3^pos^hChrdl1^+/+^ mice was evident (Fig. 6A), and the Spearman correlation coefficient (SCC) for this sequencing sample was calculated to be 0.95 (refer to Fig. 6B). The analysis revealed 3484 unique transcripts with expression levels either up-regulated or down-regulated by more than twofold in Six3^pos^hChrdl1^+/+^ compared to Six3^neg^hChrdl1^+/+^ mice. Specifically, among these, 1526 genes exhibited upregulation (depicted in red), while 1958 genes displayed downregulation (depicted in blue) (Fig. 6C). Subsequently, gene ontology analysis was employed to assess the functional categorization of transcripts in retinal development. The results indicated enrichment in categories related to neuron fate, axon development, synaptic maturation, and glia cell fate specification (Fig. 6D).

**Fig. 7:**
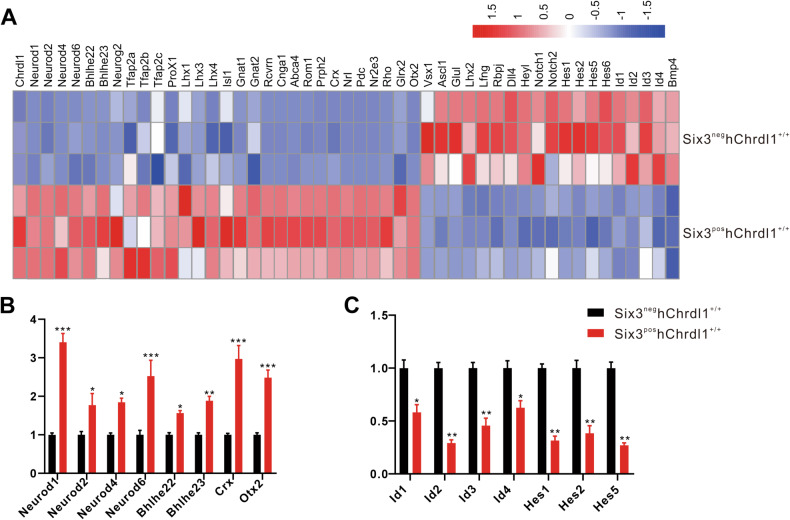
Expression analysis and qRT-PCR validation of transcription factors closely related to retinal development. **A** The heat map of expression levels for transcription factors (TFs) for retina development. **B** The qRT-PCR analyses of the up-regulated TFs genes in Six3^neg^hChrdl1^+/+^ and Six3^pos^hChrdl1^+/+^ mice at P0.5. **C** The qRT-PCR analyses of the down-regulated TFs genes in Six3^neg^hChrdl1^+/+^ and Six3^pos^hChrdl1^+/+^ mice at P0.5. Results are presented as the mean ± SEM (*n* = 3), ^*^p < 0.01, ^**^p < 0.05.

In previous studies, a suite of basic helix-loop-helix (bHLH) type transcription factors (TFs) has been implicated in the specification and differentiation of retinal neurons. We generated a heatmap illustrating the expression patterns of TFs involved in determining cell fate during retinogenesis (Fig. 7A). Consistent with the RNA-seq findings, quantitative real-time polymerase chain reaction (qRT-PCR) analysis revealed upregulation of transcriptional activators of bHLHs, including Neurod1, Neurod2, Neurod4, Neurod6, Bhlhe22, and Bhlhe23, in Chrdl1Six3-KI retinas (Fig. 7B). Conversely, transcriptional repressors of bHLHs, such as Id1, Id2, Id3, Id4, Hes1, and Hes5, exhibited a decrease in expression (Fig. 7C). These observations suggest that the conditional overexpression of Chrdl1 exerts an influence on the Chrdl1-Bmp4 pathway through the modulation of transcription factors during retinogenesis.

## Discussion

The present study represents the inaugural attempt to systematically elucidate the spatiotemporal expression patterns of *Chrdl1* and *Bmp4* in the mammalian retina. Notably, our findings reveal a distinct expression profile for *Chrdl1* and *Bmp4* in the mouse retina, deviating significantly from the dorsoventral axis pattern observed in prior non-mammalian investigations. Furthermore, we contribute novel evidence supporting the pivotal roles of *Chrdl1* and *Bmp4* in orchestrating the delicate balance between neurons and Müller glia during retinal development, as demonstrated through the construction of a conditional overexpression *Chrdl1* mouse model. These results offer fresh insights into the intricate role of *Chrdl1*-mediated *Bmp4* signaling in the developmental processes of the retina. We also comparison of Six3^pos^hChrdl1^+/+^ mouse phenotypes with the range of retinal defects previously observed in various Chrdl1and TGF-/BMP signaling component mutants provides a platform with which to evaluate the specific mechanisms by which Chrdl1 mediates Bmp4 signaling in the context of specific retinal development (refer to Supplementary Table S5).

The well-established role of *Bmp4* in promoting retinal ganglion cell survival and axon regeneration spans various stages of nervous system development [21]. During the early embryonic period, *Bmp4* plays a pivotal role in the retina, influencing processes such as lens formation and mediating apoptosis [22]. Its significance extends to the late embryonic, postnatal, and even adult stages, as exemplified in the choroid where *Bmp4* overexpression inhibits experimental neovascularization by modulating VEGF and MMP-9 [23]. This study investigates the temporal and spatial expression patterns of *Bmp4* in retinal development using immunofluorescent staining, while qRT-PCR results further elucidate *Bmp4* mRNA expression dynamics. Previous research on non-mammalian retinal development has focused on the dorsal retina as the primary site of *Bmp4* expression [8]. However, the distribution of *Bmp4* in mammalian retinal development remains poorly understood. Intriguingly, our present study systematically demonstrates, for the first time, the spatiotemporal expression patterns of *Bmp4* in mammals. This expression profile diverges significantly from the findings of previous studies in non-mammalian retinal development, providing novel insights into the distinctive role of *Bmp4* in mammalian retinal development.

*Chrdl1* is a secreted glycoprotein characterized by three cysteine-rich (CR) repeats structurally. Extensive prior research has underscored the significance of *Chrdl1* as a BMP antagonist in various systems, influencing angiogenesis [24], neural stem cell fate determination, and neurogenesis [16], as well as providing protection to the kidneys against acute and chronic injuries [25]. Additionally, *Chrdl1* has been associated with the suppression of tumor growth and metastasis [26, 27]. However, the mechanisms governing the actions of *Bmp4* and *Chrdl1*, along with their roles in retinal differentiation, remain elusive. In this study, we systematically delineate the temporal and spatial expression patterns of *Chrdl1* during mouse retinal development using RNA In situ hybridization, Immunohistochemistry and qRT-PCR. The noteworthy observation is that the expression pattern of *Chrdl1* and *Bmp4* in the mouse retina deviates significantly from the dorsoventral axis pattern documented in prior non-mammalian studies. Additionally, we identified that *Bmp4* is predominantly expressed in Sox2^+^ retinal progenitor cells (RPCs) at E14.5 and E18.5. This suggests that the *Bmp4* signal pathway may be expressed in mitotic cells and postmitotic cells at these respective stages. In order to delve deeper into the role of *Chrdl1*-mediated *Bmp4* signaling in retinal development, we generated a conditional overexpression *Chrdl1* mouse. Through analysis of the Six3^pos^hChrdl1^+/+^ mouse models, we discerned that *Chrdl1*-mediated *Bmp4* signaling plays crucial roles in maintaining a balance in the development between retinal neurons and Müller glia cells. Electroretinogram (ERG) and Optomotor Response (OMR) were also observed to deteriorate with age in mutant mice, underscoring the essential requirement for normal expression of *Chrdl1* and *Bmp4* in sustaining retinal cell structure and function. These findings suggest that *Chrdl1-*mediated *Bmp4* signaling regulates the equilibrium between retinal neurons and Müller glia in retinal development.

**Fig. 8:**
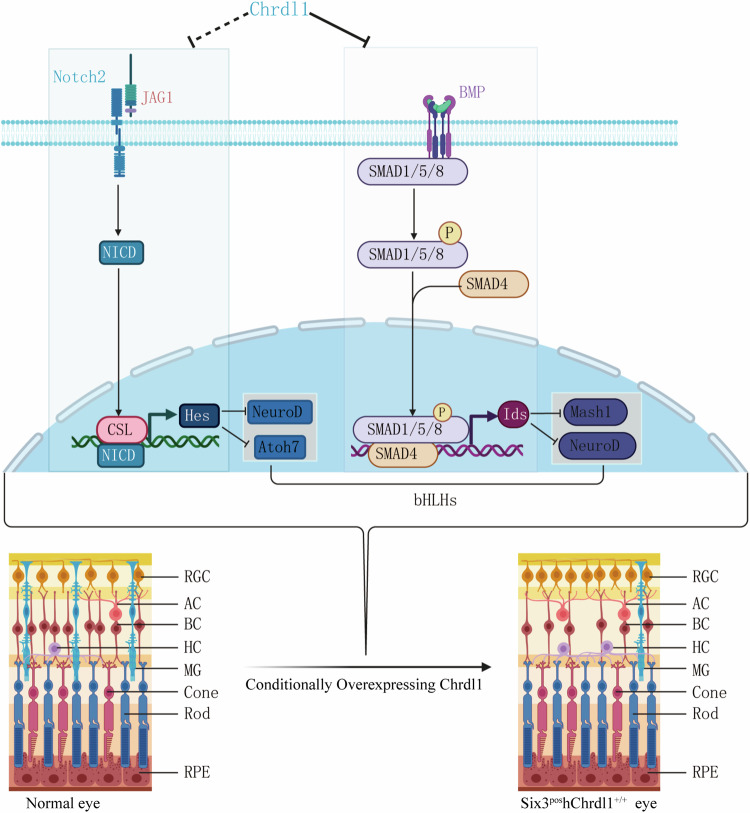
*Chrdl1* regulating retinal cell fate by bHLH transcription factor. Activation of the Notch and BMP signaling pathways leads to increased levels of Hes and Id proteins, which inhibit neuronal differentiation by binding and inhibiting bHLH transcription factor activity. Chrdl1 reverses this process, promoting neuronal differentiation and inhibiting gliogenesis.

During retinal ontogenesis, the differentiation of each retinal cell type necessitates the orchestrated activation of a specific ensemble of transcription factors (TFs) to induce their commitment to a particular cellular fate. Notably, the bHLHs and homeodomain families represent pivotal TFs in this regulatory paradigm [28–32]. Concurrently, in the context of central nervous system (CNS) development, the neurogenic bHLHs are susceptible to antagonism by Inhibitor of DNA binding and differentiation (Ids) proteins [33–35]. Ids have been substantiated as canonical early response genes in BMPs signaling across diverse cellular contexts [36, 37]. The promoter region of the Ids gene has been previously elucidated to harbor a BMP response element (BRE), recognized by a Smad1/5-Smad4 complex in response to BMPs, thereby eliciting downstream gene transcription [38]. Based on the findings from RNA-seq analysis, we posit that the regulatory influence of *Bmp4* on retinal development may be mediated by Ids, which has the capacity to deactivate bHLH TFs and impede neuronal differentiation. In the course of our investigation, the expression levels of Ids isoforms (Id1, Id2, Id3, Id4) exhibited a noteworthy reduction subsequent to *Chrdl1* overexpression. Concurrently, the expression levels of diverse bHLH TFs implicated in the regulation of retinal neuron differentiation were significantly elevated. Consequently, we postulate that the heightened expression of *Chrdl1* impedes the regulatory dynamics of the BMP-Ids pathway during retinogenesis (Fig. 8), yielding three discernible outcomes: (i) augmentation in the expression of bHLH TFs, a paramount regulatory factor in retinal development, expediting the differentiation of diverse retinal neurons and culminating in an augmented population of early-born neurons; (ii) concomitant reduction in the number of retinal progenitor cells (RPCs) subsequent to *Bmp4* inhibition, thereby impacting bipolar cell (BC) differentiation; and (iii) attenuation of BMPs signaling by *Chrdl1*, thereby inhibiting its facilitative role in glial differentiation and consequently diminishing the generation of Müller glial cells.

In addition to the Ids family, the downstream effector Hes family within the Notch signaling pathway serves as an additional antagonist to neurogenic bHLH transcription factors [39]. Hes1 and Hes5 emerge as pivotal transcriptional regulators essential for the maintenance of retinal progenitor cells (RPCs). Notably, studies have demonstrated that the overexpression of Hes1 and Hes5 exerts a discernible influence on the fate determination of Müller glia and RPCs during retinogenesis, compromising neuronal differentiation [40–42]. In the course of our investigation, we observed a downregulation in the expression levels of multiple effectors downstream of the Notch pathway, including Hes1 and Hes5, consequent to the overexpression of *Chrdl1*. Consequently, it is conjectured that Chrdl1 engages in molecular interactions with the Notch pathway (Fig. 8). It is imperative to acknowledge certain limitations inherent in this study. The potential contrasting alterations resulting from the deficiency of *Chrdl1* remain unclear and warrant further exploration. Subsequently, the utilization of a *Chrdl1* knockout model is imperative to ascertain whether such deficiency will precipitate a reduction in neuronal differentiation and a concomitant augmentation in Müller glial differentiation.

In summation, within the context of retinogenesis, *Chrdl1*, serving as an antagonist to *Bmp4*, exhibits the capacity to intricately modulate the fate commitment of progenitor cells. This modulation is achieved through the specific binding interactions with target mRNAs, namely, Id1/2/3/4, Hes1/5, Neurod1/2/4, and Bhlhe22/23, thereby indirectly regulating the expression levels of transcription factors (TFs), notably bHLHs and homeodomain families. Concurrently, *Chrdl1* engages in interactions with the Notch pathway, as depicted in Fig. 8. Subsequent to the perturbation in TF levels induced by the conditional overexpression of *Chrdl1*, an excess of *Chrdl1* is observed to impede gliogenesis while concurrently facilitating the differentiation of retinal progenitor cells (RPCs) into retinal neurons. This dysregulation precipitates the ultimate disruption in the amplitude of electroretinogram (ERG) responses and visual acuity. Consequently, our investigations contribute to an enhanced comprehension of the nuanced role played by *Chrdl1*-mediated *Bmp4* signaling in retinal development. Furthermore, our findings present a novel conceptual framework with potential implications for the therapeutic management of *Chrdl1*-mediated *Bmp4* signaling-associated ocular pathologies.

## Materials and methods

### Animals

*C57BL/6JGpt-Rosa26*^*em(hChrdl1)*^ (Rosa26-hChrdl1Six3-Cre) mice were generated as follows. We generated a conditional loxP-STOP-human Chrdl1 knock-in mouse line under the control of the ubiquitous Rosa26 promoter on a C57Bl6 background. Homozygous animals were bred with SiX3-Cre animals, to promote expression of human Chrdl1 in retina. Six3-expressing F1 animals were viable and bred again with homozygous conditional animals, to generate animals carrying 2 alleles of the knocked-in human Chrdl1 (Fig. S1A). Six3^neg^ hChrdl1^+/+^ animals were used as controls.

The breeding and euthanasia procedures involving experimental murine subjects were executed with the explicit endorsement of the Animal Ethics Committee situated within the Laboratory Animal Center at Wuhan University (Ethical Approval Number: MRI2021-LACJ029), in strict accordance with the guidelines stipulated by the National Institutes of Health (NIH). The mice were diligently housed within the confines of Specific Pathogen-Free (SPF) conditions, meticulously maintained at the Animal Experimental Center of Renmin Hospital, Wuhan University, wherein ambient temperature was rigorously upheld within the range of 24–26 °C, and humidity levels were maintained within the parameters of 40–60%. Mice were afforded ad libitum access to both water and standard rodent feed. For the precise determination of gestational age in embryonic murine cohorts, the assessment of mating status was discerned through vigilant observation of the presence of the vaginal plugs, with an observational margin of error not exceeding 6 h. The identification of a vaginal plug served as the demarcation point for embryonic day 0.5 (E0.5). Genotyping procedures for all subjects within the laboratory animal cohort were systematically conducted via standard Polymerase Chain Reaction (PCR) methodologies (Fig. S1B), and the pertinent primer sequences employed for this purpose are detailed in Table S1.

### Immunofluorescence

The sections were subjected to an overnight incubation at 4 °C with primary antibodies (refer to Supplementary Table S2) and secondary antibodies (refer to Supplementary Table S3) for 1 h. Following this immunostaining procedure, 4’,6-diamidino-2-phenylindole (DAPI) was employed for nuclear staining, and the specimens were imaged using a confocal laser microscope (Zeiss LSM880, Germany).

### Real-Time Quantitative Reverse Transcription (qRT)-PCR

After euthanizing the mice, total RNA was promptly extracted from the retinas of various genotypes using the TRIzol reagent (Invitrogen, Carlsbad, CA, USA). Gene transcription levels were quantified through qRT-PCR employing the SYBR Green I fluorescent dye (Vazyme Biotech Co., Ltd, Nanjing, China). All values were standardized relative to the endogenous levels of Gapdh and normalized to those of the control group. The primer sequences utilized for qRT-PCR are delineated in Supplementary Table S4.

### RNA Sequencing Analysis (RNA-seq)

After extracting RNA from P0.5 retinas using TRIzol Reagent (Invitrogen, 10296028), DNaseI treatment was applied for DNA digestion. The quality and integrity of the RNA were evaluated using the A260/A280 ratio (Thermo Fisher, USA) and agarose gel electrophoresis, respectively. Subsequently, a stranded RNA sequencing library was prepared with 2 g total RNAs following the manufacturer’s instructions for Illumina® (Seqhealth Co., Ltd. DR08402). PCR products in the range of 200–500 bps were enriched, quantified, and sequenced using the PE150 model (Illumina, USA). Raw sequencing data underwent initial filtration using Trimmomatic v0.36. The clean data were then aligned to the retina reference genome using STRA software with default parameters. RPKMs were calculated following featureCounts, which counted reads corresponding to each gene’s exon regions. The identification of differentially expressed genes between groups was conducted using the edgeR program v3.12.1. Statistical analysis of these genes involved Gene Ontology analysis and Kyoto Encyclopedia of Genes and Genomes enrichment analysis (KOBAS v2.1.1). To determine statistical differences in gene expression, a *p*-value cutoff of 0.05 and a fold-change cutoff of 2 were applied.

### RNA in situ hybridization

Sections were desiccated in a hybridization oven at 50 °C for 15 min, fixed with 4% paraformaldehyde (PFA) for 20 min at room temperature, and permeabilized with 2 μg/mL proteinase K in phosphate-buffered saline (PBS) at room temperature for 10 min. Prior to hybridization, the sections underwent acetylation in 0.25% acetic anhydride for 10 min. Subsequently, the sections were incubated with a digoxigenin-labeled probe (0.2 ng/μL) diluted in hybridization buffer (50% deionized formamide, 5 × SSC, 5 × Denhart’s, 250 μg/mL tRNA, and 500 μg/mL Herring sperm DNA) beneath coverslips in a hybridization oven at 65 °C overnight. The following day, the sections were subjected to four washes with 0.1 × SSC for 20 min each at 65 °C. Following this, they were treated with 20 μg/mL RNase A at 37 °C for 20 min and subsequently blocked in 10% normal sheep serum at room temperature for 3.5 h. Slides were then incubated with an anti-digoxigenin-alkaline phosphatase (AP) conjugated antibody (Roche) diluted 1:5000 at 4 °C overnight. The chromogenic substrate BCIP/NBT (Roche) was employed for visualization.

### Electroretinogram (ERG)

The Electroretinogram procedures followed previous methodologies [43], utilizing the RetiMINER IV system (IRC Medical Equipment, Chongqing, China) for measuring ERG responses in mice. The experimental subjects included P30, P90, and P240 Chrdl1 Six3^pos^hChrdl1^+/+^ mice, as well as Six3^neg^hChrdl1^+/+^ mice. Typically, the target mice were acclimated in a dark room for 4 – 6 h prior to the experiment. Subsequently, the mice were exposed to varying intensities of Ganzfeld illumination: 0.0001, 0.0003, 0.01, 0.03, 0.1, 0.3, 1.0, 3.0, and 10 cd·e;s/m2. Following this exposure, the mice underwent light adaptation for 5 min on a saturated background, followed by obtaining optical position recordings under five levels of stimulation (0.3, 1.0, 3.0, 10, 30 cd·e;s/m2). The Flash stimuli throughout the experiment were generated by a Xenon lamp.

### Optomotor Response (OMR)

To assess the visual acuity of mice, Optomotor Response (OMR) recordings were conducted following established protocols [44]. In brief, OMR recordings for P30, P90 and P240 Six3^pos^hChrdl1^+/+^ mice, as well as Six3^neg^hChrdl1^+/+^ mice, were obtained under uniform conditions. Following adaptation to darkness, mice were freely tested on a platform positioned at the center of a box, which featured four identical LED screens displaying rotating vertical black and white stripes. Ten spatial frequencies ranging from 0.1 to 0.6 cycles/deg were systematically examined. The movements of the mice were recorded using an infrared camera placed above the chamber to quantify the number of head tracking movements during each spatial frequency test.

### Statistical analysis

Each experiment was conducted with a minimum of three repetitions, and the final data was represented as the mean ± SEM. Statistical analysis was carried out using GraphPad Prism 6.0, employing the unpaired *t*-test for the analysis of original values. A significance threshold of *p* < 0.05 was applied, indicating statistical significance when met.

### Supplementary information

SUPPLEMENTAL MATERIAL

## Data Availability

All data supporting the findings of this study are available within the paper and its Supplementary Information.
