# Supplementary material for: Chrdl1-mediated BMP4 inhibition disrupts the balance between retinal neurons and Müller Glia
Source: Cell Death Discov. 2024 Aug 17;10:367. doi: 10.1038/s41420-024-02129-6 (PMC11329631; doi:10.1038/s41420-024-02129-6)
Supplement: Supplementary file 1 — SUPPLEMENTAL MATERIAL [file 41420_2024_2129_MOESM1_ESM.docx]

**Supplementary Materials:**

**
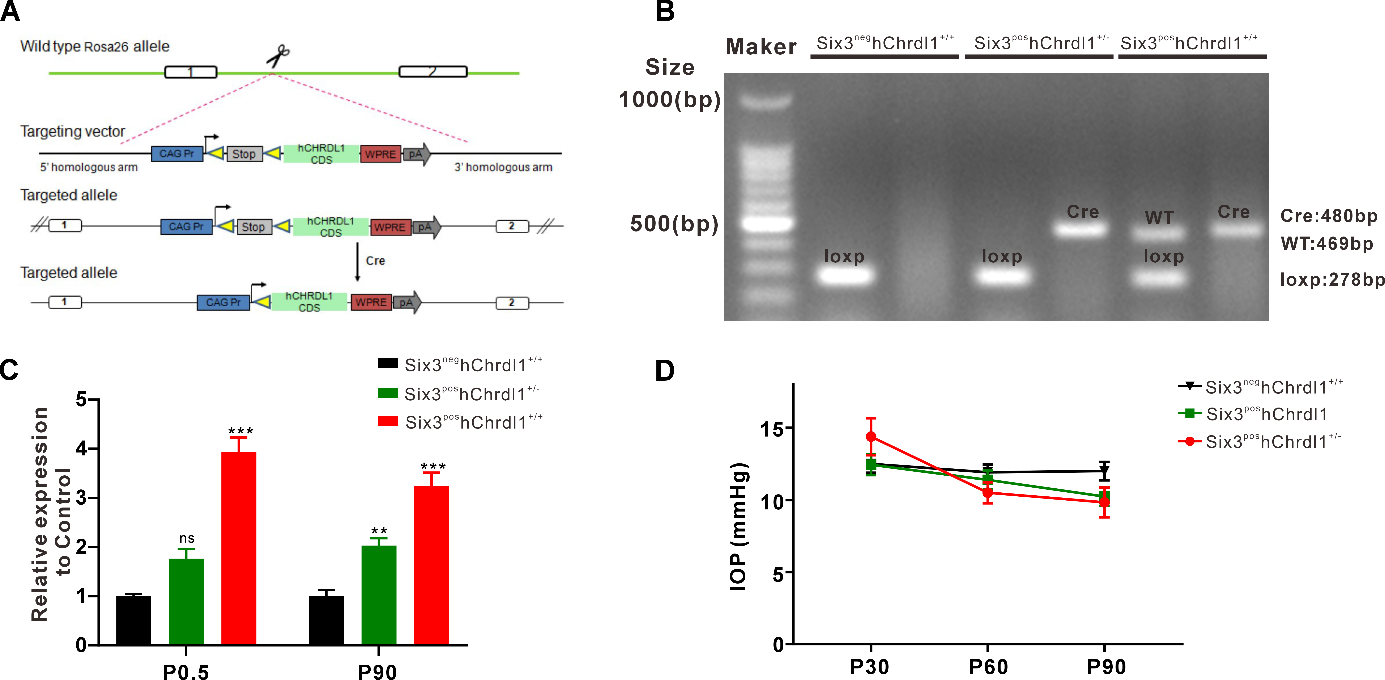
Figure S1. The establishment of *Chrdl1* overexpression model.** (A) A floxed *Chrdl1* mouse line was bred with the Six3-Cre transgenic mice to conditionally overexpress *Chrdl1* by Rosa26 in the developing retina. (B) All the genotypes (Six3^neg^hChrdl1^+/+^, Six3^pos^hChrdl1^+/-^ and Six3^pos^hChrdl1^+/+^) were determined by PCR. (C) Increased expression of *Chrdl1* in the Six3^pos^hChrdl1^+/+^ retina. Results are presented as the mean ± SEM (*n* = 3). ^*^ *p* < 0.01, ^**^ *p* < 0.05, ^***^ *p* < 0.001.


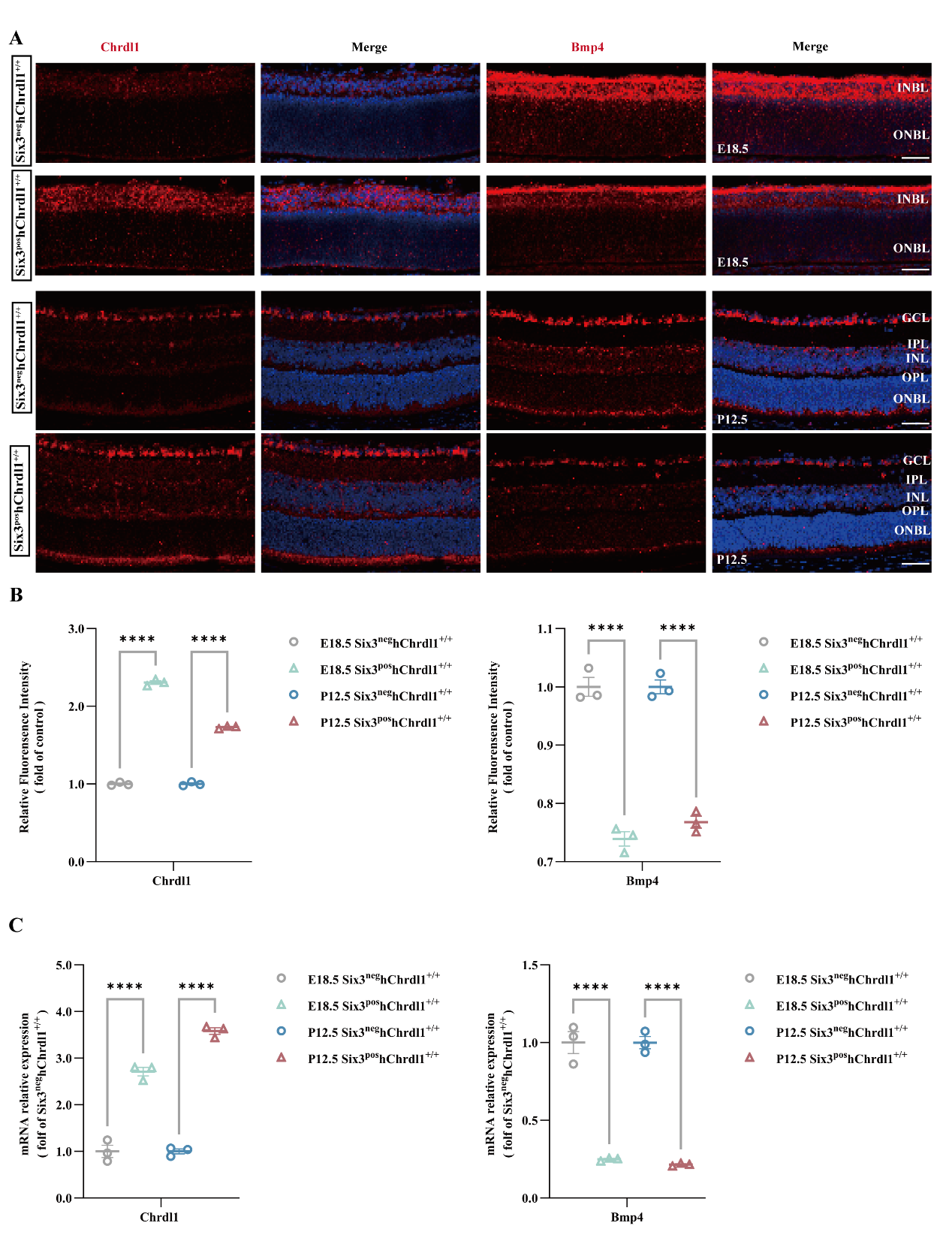


**Figure S2. Validation of the efficacy of the Chrdl1 overexpression model in inhibiting BMP4 expression.** (A) Sections from different time points (E18.5 and P12.5) Six3^neg^hChrdl1^+/+^ and Six3^pos^hChrdl1^+/+^ retinas were immunofluorescence with antibodies (Chrdl1 and Bmp4) and weakly counterstained with nuclear DAPI. Red represents Chrdl1 and Bmp4, respectively. Scale = 20 μm. (B) Quantitative assessment of the relative fluorescence intensity in Figure 2A. (C) The mRNA expression levels of Chrdl1 and BMP4 at E18.5 and P12.5 in Six3^neg^hChrdl1^+/+^ and Six3^pos^hChrdl1^+/+^ retinas were determined by qRT-PCR analysis. Results are presented as the mean ± SEM (*n* = 3). ^****^ *p* < 0.000

**
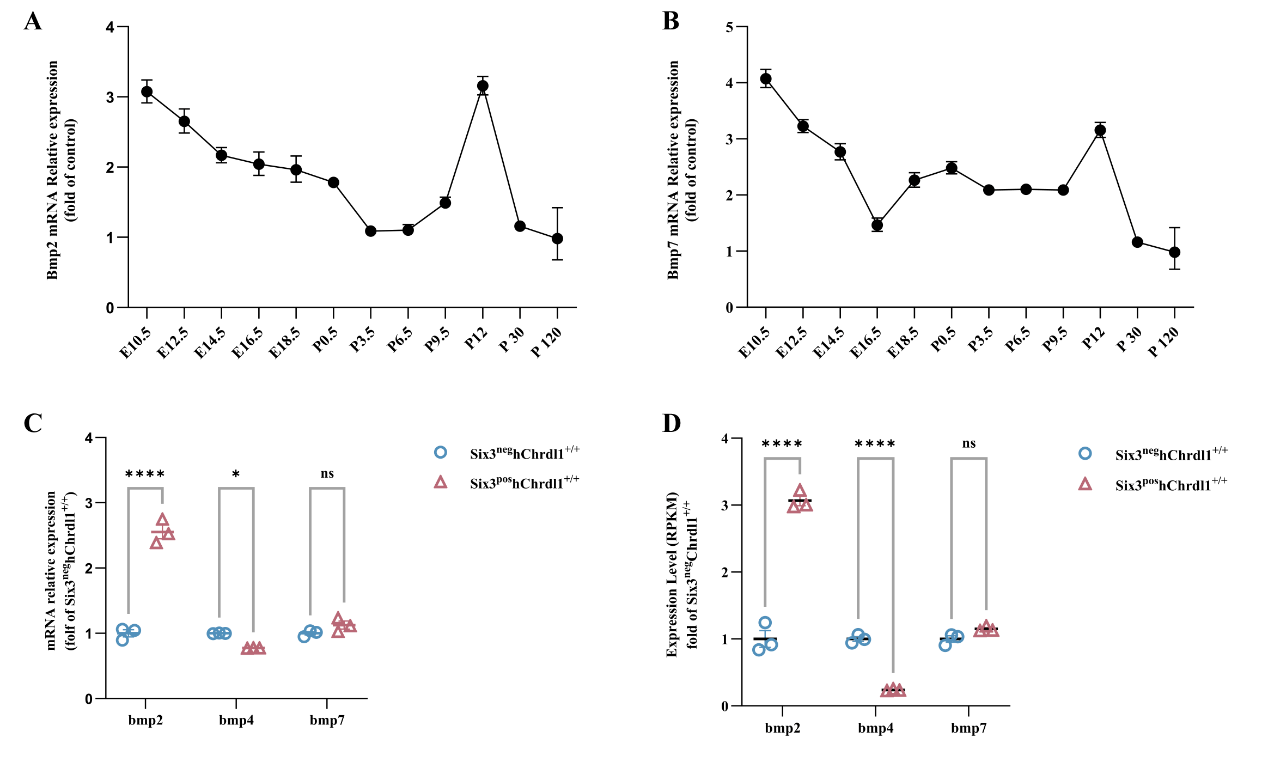
**

**Figure S3. The Impact of Chrdl1 Overexpression on the Transcriptional Regulation of BMPs.** (A) Temporal expression levels of *Bmp2* from embryonic to adult stages during retinal development as determined by qRT-PCR analysis. (B) Temporal expression levels of *Bmp2* from embryonic to adult stages during retinal development as determined by qRT-PCR analysis. (C) The mRNA expression levels of BMP2/4/7 in Six3^neg^hChrdl1^+/+^ and Six3^pos^hChrdl1^+/+^ retinas were determined by qRT-PCR analysis. Results are presented as the mean ± SEM (*n* = 3). (D) The RPKM levels of BMP2/4/7 in Six3^neg^hChrdl1^+/+^ and Six3^pos^hChrdl1^+/+^ retinas were determined by RNA-seq analysis. Results are presented as the mean ± SEM (*n* = 3). ^****^ *p* < 0.0001, ^*^ *p* < 0.05.

**
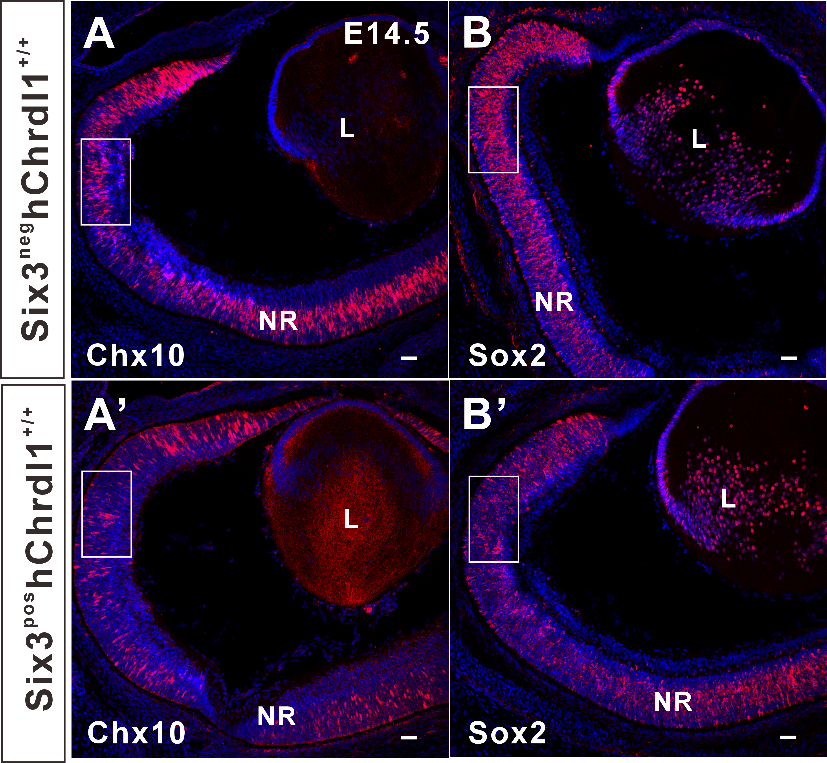
**

**Figure S4. Effect of *Chrdl1* overexpression on the retina progenitor proliferation.** (A-B, A’-B’) Immunostaining of E14.5 Six3^neg^hChrdl1^+/+^ and Six3^pos^hChrdl1^+/+^ retinal sections with antibodies against Chx10 or Sox2. All sections were weakly counterstained with nuclear DAPI. Scale=20 μm.


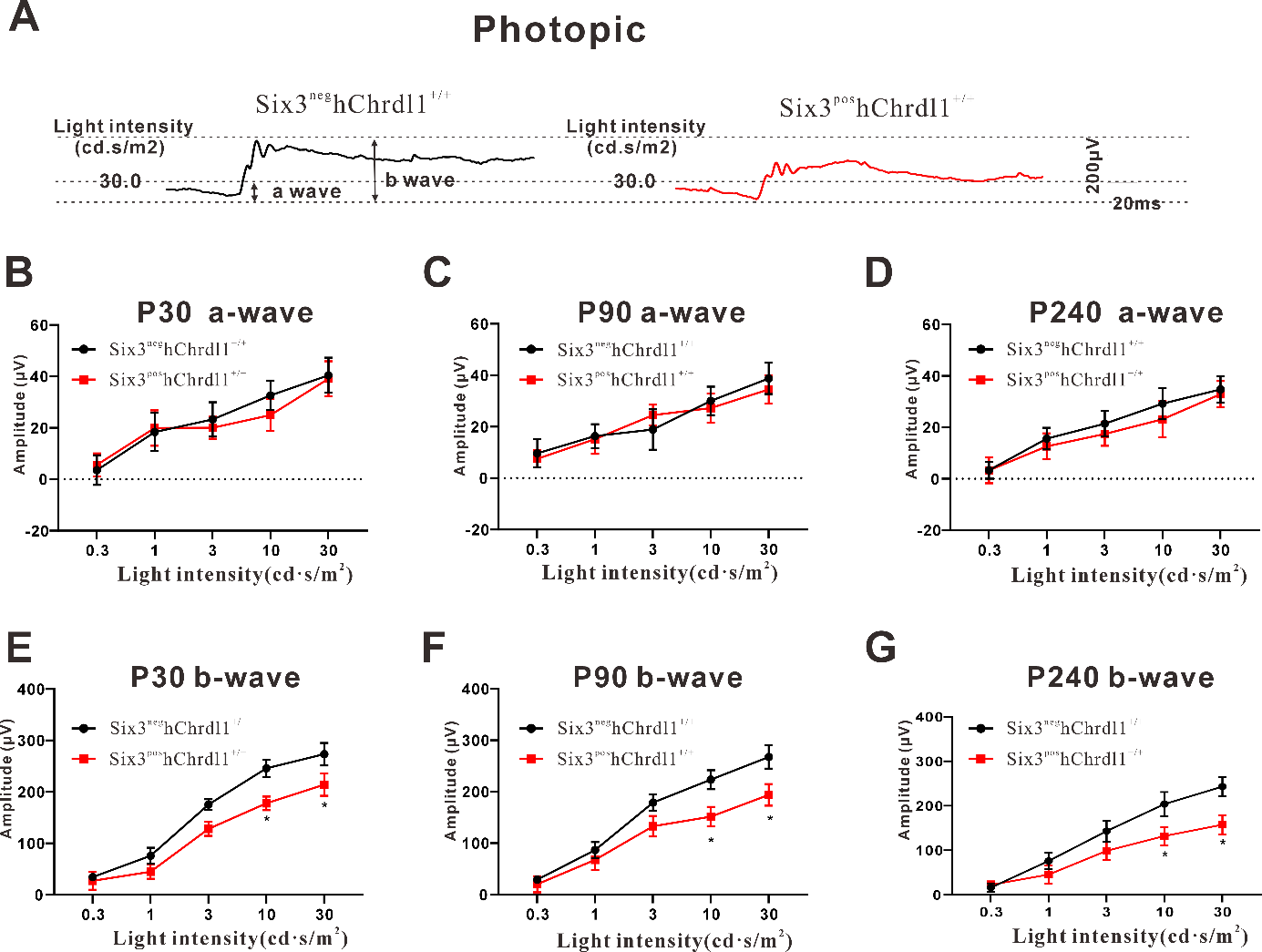


**Figure S5. *Chrdl1* overexpression causes a deficit in electroretinogram (ERG) responses and visual acuity.** (A) Representative ERG waveforms of 30.0 light intensity (cdꞏs/m^2^) from light-adapted Six3^neg^hChrdl1^+/+^ and Six3^pos^hChrdl1^+/+^ aged P30. (B-D) The amplitudes of the photopic ERG a-waves elicited from mice were recorded at P30, P90, and P240. (E-G) The amplitudes of the photopic ERG b-waves elicited from mice were recorded at P30, P90, and P240. Results are presented as the mean ± SEM (*n* = 12). ^*^ *p* < 0.01.

**Table S1 Primer sequences for genotyping used in this study**

| Gene | Forward primer | Reverse primer |
| --- | --- | --- |
| Rosa-WT | AGTCGCTCTGAGTTGTTATCAG | TGAGCATGTCTTTAATCTACCTCGATG |
| Rosa-Mut | AGTCGCTCTGAGTTGTTATCAG | GTCAATGGAAAGTCCCTATTGGCGT |
| Six3-cre | TCGATGCAACGAGTGATGAG | TTCGGCTATACGTAACAGGG |

**Table S2 Primary antibodies used in this study**

| Antibody | Company | Catalog No. | Working dilution | host |
| --- | --- | --- | --- | --- |
| AP2α | Abcam | ab108311 | 1:1000 | Rb |
| Brn3a | Synaptic System | 411003 | 1:1000 | Rb |
| BMP4 | Abcam | ab39973 | 1:500 | Rb |
| Chrdl1 | Thermo Fisher | PA578591 | 1:100 | Rb |
| Calbindin | SWANT | cb38 | 1:5000 | Rb |
| Caspase3 | R&D | AF835- | 1:5000 | Rb |
| Chat | Millipore | AB144P | 1:200 | Gt |
| Chx10 | Exalpha | X1180P | 1:500 | sheep |
| Ki67 | Invitrogen | MA5-14520 | 1:1000 | Rb |
| Opsin Red/Green | Millipore | AB5405 | 1:1000 | Rb |
| Pax6 | Millipore | ab2237 | 1:2000 | Rb |
| PCP-2 | Santa cruz | sc-49072 | 1:1000 | Gt |
| PKCα | Santa cruz | SC-208-G | 1:500 | Gt |
| PKCα | Sigma | P4334 | 1:1000 | Rb |
| Rbpms | Phosphosolutions | 1830 | 1:500 | Rb |
| Recoverin | Millipore | ab5585 | 1:5000 | Rb |
| Rhodopsin | Millipore | MAB5356 | 1:500 | Ms |
| Sox2 | R&D | AF2018 | 1:500 | Gt |
| Sox9 | Millipore | ab5535 | 1:200 | Rb |
| Tuj1 | Covance | MMS-435P | 1:1000 | Ms |

**Table S3 Secondary antibodies used in this study**

| Antibody | Company | Catalog No. | Dilution |
| --- | --- | --- | --- |
| Alexa Fluor® 594 AffiniPure Donkey Anti-Rabbit IgG | Jackson | 711585152 | 1:500 |
| Alexa Fluor® 488 AffiniPure Donkey Anti-Rabbit IgG | Jackson | 711545152 | 1:500 |
| Alexa Fluor® 594 AffiniPure Donkey Anti-Mouse IgG | Jackson | 715585151 | 1:500 |
| Alexa Fluor® 488 AffiniPure Donkey Anti-Mouse IgG | Jackson | 715545151 | 1:500 |
| Alexa Fluor® 594 AffiniPure Donkey Anti-Sheep IgG | Jackson | 713585147 | 1:500 |
| Alexa Fluor® 488 AffiniPure Donkey Anti-Sheep IgG | Jackson | 713545147 | 1:500 |
| Alexa Fluor® 594 AffiniPure Donkey Anti-Goat IgG | Jackson | 705585147 | 1:500 |
| Alexa Fluor® 488 AffiniPure Donkey Anti-Goat IgG | Jackson | 705545147 | 1:500 |

**Table S4 Primer sequences for qRT-PCR used in this study**

| Gene | | Forward primer | Reverse primer |
| --- | --- | --- | --- |
| Neurod1 | | ATGACCAAATCATACAGCGAGAG | TCTGCCTCGTGTTCCTCGT |
| Neurod2 | | AAGCCAGTGTCTCTTCGTGG | GCCTTGGTCATCTTGCGTTT |
| Neurod4 | | AGCTGGTCAACACACAATCCT | TTCCATAAGAGCCCGGTCTTC |
| Neurod6 | | ACGACTGGAAAGGGTCAAGTT | GACCACTTTTCGCAAATTGTCC |
| BHLHe22 | | CGCCTCAACATCAACGCTC | TGCGCGTAAGGAATGACCG |
| BHLHe23 | | GCGCTTGGTAGCATACCTCAA | CCGAGAACGGGTAAATCGCT |
| BMP4 | | ATTCCTGGTAACCGAATGCTG | CCGGTCTCAGGTATCAAACTAGC |
| Crx | | GTTCAAGAATCGTAGGGCGAA | GGACTCCAAATGGACACGGT |
| Chrdl1 | | AACCTCCAAGCCAAAACTTTGA | CCAGTGCTACTTTTCTGGTTGTC |
| Otx2 | | TATCTAAAGCAACCGCCTTACG | GCCCTAGTAAATGTCGTCCTCTC |
| Id1 | | CCTAGCTGTTCGCTGAAGGC | GTAGAGCAGGACGTTCACCT |
| Id2 | | TCCGGTGAGGTCCGTTAGG | CAGACTCATCGGGTCGTCC |
| Id3 | | CTGTCGGAACGTAGCCTGG | GTGGTTCATGTCGTCCAAGAG |
| Id4 | | CAGTGCGATATGAACGACTGC | GACTTTCTTGTTGGGCGGGAT |
| Hes1 | | TCAACACGACACCGGACAAAC | ATGCCGGGAGCTATCTTTCTT |
| Hes2 | | CGGATCAACGAGAGCCTAAGC | TAGGAAGCGCACAGTCATTTC |
| Hes5 | | AGTCCCAAGGAGAAAAACCGA | GCTGTGTTTCAGGTAGCTGAC |
| Ap2α | | ACGACCCCTACAGCCTGAAT | GGCGTGAGGTAAGGAGTGG |
| Calbindin | GGCTTCATTTCGACGCTGAC | | ACGTGAGCCAACTCTACAATTC |
| Chx10 | AAGCCCAAATCCGAGACAGTG | | CGCAGCTAACAAATGCCCA |
| Cx57 | | AATTTACTGGGTGGCATCCTAGA | AGTACCAGCATTCGGAAGATGA |
| GFAP | | CGGAGACGCATCACCTCTG | TGGAGGAGTCATTCGAGACAA |
| GS | | GTTCCCACTTGAACAAAGGCA | ACCCAGATATACATGGCTTGGA |
| Opn1sw | | CAGCCTTCATGGGATTTGTCT | GTCGCAACTTTTTGTAATGCAGT |
| Pax6 | | GTTGTGTGAGTAAAATTCTGGGC | GAGTCGCCACTCTTGGCTTA |
| PKCα | | GTTTACCCGGCCAACGACT | TCTTTCACCTCATGCACGTTC |
| Rcvrn | | ACGACGTAGACGGCAATGG | CCGCTTTTCTGGGGTGTTTT |
| Rhodopsin | | CCCTTCTCCAACGTCACAGG | GTAGAGCGTGAGGAAGTTGATG |
| Sox2 | | GCGGAGTGGAAACTTTTGTCC | GGGAAGCGTGTACTTATCCTTCT |
| Sox9 | | AGTACCCGCATCTGCACAAC | ACGAAGGGTCTCTTCTCGCT |
| Rbpms | | GCCTACGGGCTGTTTAGATGT | CCGGACCCTATTTGTCAGCG |

Table S5 Comparison of Mice Models Related to Chrdl1/Bmp4

| Animal model | Ocular phenotype | Variations in cellularity (retina) | Proliferation and apoptosis | Transcription factor | PMID |
| --- | --- | --- | --- | --- | --- |
| caBmpr1a/b | Abnormal Retinal Development | Bipolar cells (↑)  Müller cells（↑） | No effect | Id1 (↑)  Hey2 (↓) | 24890415 |
| dnBmpr1a/b | Abnormal Retinal Development | Bipolar cells (↓)  Müller cells（↓） | No effect | Id1 (↓)  Hey2 (↑) | 24890415 |
| Smad4 cKO mice | Microphthalmia deformity | RGC (↓)  Rod photoreceptor(↓) | Transient Enhancement of Apoptosis (E12.5) | N.A | 21273545 |
| Chrdl1 KO | anterior chamber (shallower) cornea (thicker) | RGC (No change) | N.A | N.A | 32452990 |
| Six3^pos^hChrdl1^+/+^ mice | Abnormal Retinal Development | Early-Born Neurons (↑)  later-born neurons (↓) | No effect | bHLH TFs (↑)  Ids(1/2/3/4) (↓) | N.A |
